# Incidence of contrast-associated acute kidney injury: a prospective cohort

**DOI:** 10.1590/2175-8239-JBN-2023-0019en

**Published:** 2023-09-25

**Authors:** André Lucas Ribeiro, Fabricio Bergelt de Sousa, Beatriz Cavalcanti Juchem, André Zimerman, Guilherme Bernardi, Manoela Astolfi Vivan, Tiago Severo Garcia

**Affiliations:** 1 Hospital de Clínicas de Porto Alegre, Porto Alegre, RS, Brazil.; 2 Harvard Medical School, Brigham & Women's Hospital, TIMI Study Group, Boston, Massachusetts United States.

**Keywords:** Contrast Media, Acute Kidney Injury, Contrast-associated Acute Kidney Injury, Computed Tomography, Meios de Contraste, Injúria Renal Aguda, Lesão renal aguda associada ao contraste, Tomografia Computadorizada

## Abstract

**Introduction:**

Contrast-associated acute kidney injury (CA-AKI) is a deterioration of kidney function that occurs after the administration of a iodinated contrast medium (ICM). Most studies that defined this phenomenon used older ICMs that were more prone of causing CA-AKI. In the past decade, several articles questioned the true incidence of CA-AKI. However, there is still a paucity of a data about the safety of newer ICM.

**Objective:**

To assess the incidence of CA-AKI in hospitalized patients that were exposed to computed tomography (CT) with and without ICM.

**Methods:**

Prospective cohort study with 1003 patients who underwent CT in a tertiary hospital from December 2020 through March 2021. All inpatients aged > 18 years who had a CT scan during this period were screened for the study. CA-AKI was defined as a relative increase of serum creatinine of ≥ 50% from baseline or an absolute increase of ≥ 0.3 mg/dL within 18 to 48 hours after the CT. Chi-squared test, Kruskal-Wallis test, and linear regression model with restricted cubic splines were used for statistical analyses.

**Results:**

The incidence of CA-AKI was 10.1% in the ICM-exposed group and 12.4% in the control group when using the absolute increase criterion. The creatinine variation from baseline was not significantly different between groups. After adjusting for baseline factors, contrast use did not correlate with worse renal function.

**Conclusion:**

The rate of CA-AKI is very low, if present at all, with newer ICMs, and excessive caution regarding contrast use is probably unwarranted.

## RESUMO

**Introdução:**

Lesão renal aguda associada ao contraste (LRA-AC) é uma deterioração da função renal que ocorre após a administração de meio de contraste iodado (MCI). A maioria dos estudos que definiram esse fenômeno utilizaram MCI mais antigos, mais propensos a causar LRA-AC. Na última década, diversos artigos questionaram a verdadeira incidência de LRA-AC. Entretanto, ainda há escassez de dados sobre a segurança dos MCI mais novos.

**Objetivo:**

Avaliar a incidência de LRA-AC em pacientes hospitalizados expostos à tomografia computadorizada (TC) com e sem MCI.

**Métodos:**

Estudo de coorte prospectivo com 1.003 pacientes submetidos a TC em hospital terciário, de dezembro/2020 a março/2021. Todos os pacientes internados com idade ≥ 18 anos que realizaram TC nesse período foram selecionados. A LRA-AC foi definida como aumento relativo de creatinina sérica de ≥ 50% em relação ao valor basal ou aumento absoluto de ≥ 0,3 mg/dL dentro de 18 a 48 horas após a TC. Utilizamos o teste qui-quadrado, teste de Kruskal-Wallis e modelo de regressão linear com splines cúbicos restritos para análises estatísticas.

**Resultados:**

A incidência de LRA-AC foi 10,1% no grupo exposto ao MCI e 12,4% no grupo controle ao usar o critério de aumento absoluto. A variação da creatinina em relação ao valor basal não foi significativamente diferente entre os grupos. Após ajuste para fatores basais, o uso de contraste não se correlacionou com pior função renal.

**Conclusão:**

A taxa de LRA-AC é muito baixa, caso exista, com MCIs mais novos, e a cautela excessiva quanto ao uso de contraste provavelmente não se justifica.

## Introduction

Contrast-associated acute kidney injury (CA-AKI) is a sudden deterioration in renal function that occurs shortly after the administration of iodinated contrast medium (ICM)^1^. Historically, high-osmolality ionic contrast agents were associated with a higher risk of CA-AKI compared to more recent low-osmolality or iso-osmolality nonionic agents^2^. A seminal study comparing these contrast agent types reported a 7% incidence of CA-AKI with meglumine/sodium diatrizoate (ionic contrast agent) and 3% with iohexol (nonionic contrast agent), with a higher risk observed in patients with a history of chronic kidney disease (CKD) and diabetes mellitus (DM), as well as in those exposed to greater contrast volumes^3^. Barrett et al.^4^ also prospectively compared different contrast media by examining CA-AKI incidence in patients with CKD (baseline serum creatinine [SCr] ≥ 1.5 mg/dL) after exposure to either iopamidol-370 or iodixanol-320, identifying a similarly low rate of 4% in both groups.

More recently, McDonald et al.^5,6^ have published a series of studies questioning the existence of CA-AKI in patients with and without CKD, including CKD stages IV and V, and found no increased risk of CA-AKI after propensity score stratification. In addition, McDonald et al.^7^ recently published a meta-analysis of 25,950 patients, revealing comparable incidences of acute kidney injury (AKI) in patients exposed or not to ICM (6.4% and 6.5%, respectively). Additionally, registry studies and cohorts of hospitalized patients have shown that some degree of renal insufficiency is expected to develop in patients admitted to tertiary hospitals, as evidenced by Nash et al.’s^8^ analysis of 4,622 patients, 7.2% of whom developed some degree of AKI.

Consequently, it is of utmost importance to better understand the true incidence of CA-AKI. Given the limitations of most studies on this topic due to their retrospective nature, we aimed to conduct a prospective cohort study to determine the incidence of CA-AKI in a tertiary hospital setting. Our study stratified the results according to the risk factors most commonly cited in the literature to provide a more comprehensive understanding of CA-AKI and its implications for the use of contrast media in clinical practice^9^.

## Methods

We conducted a prospective cohort study between December 2020 and March 2021 to assess the incidence of CA-AKI in a tertiary hospital in Porto Alegre, Brazil. In Brazil, tertiary hospitals are specialized centers that manage complex and severe cases, offering advanced diagnostic and therapeutic services, and are usually affiliated with academic institutions for research and training purposes. The definition of CA-AKI was based on the Kidney Disease Improving Global Outcomes (KDIGO) guidelines and defined as a relative increase of SCr of ≥ 50% from baseline or an absolute increase of ≥ 0.3 mg/dL within 18 to 48 hours following a computed tomography (CT) scan. CKD definition and classification also adhered to KDIGO definitions^10^.

Baseline creatinine was defined using the last creatinine value before the CT scan, and post-CT creatinine values were obtained within the established 18 to 48-hour timeframe. The decision whether to perform a contrasted exam was made collaboratively by the attending physician and the radiology department.

Inclusion criteria were all patients undergoing a CT scan, either enhanced with ICM or unenhanced, within the specified time period. Exclusion criteria were patients with missing data (e.g., no baseline creatinine or post-CT creatinine within 18 to 48 h), patients already on dialysis, patients who underwent multiple CT scans, patients who had surgery between SCr blood sample collections, and patients who had angiography instead of contrasted CT scan.

The primary outcome was the estimated effect of contrast use in post-CT renal function, as assessed by a linear regression model adjusted for age, sex, baseline creatinine, DM, hypertension, and use of furosemide or nonsteroidal anti-inflammatories (NSAIDs). Secondary outcomes included incidence of CA-AKI stratified by CKD stages (IIIa, IIIb, IV, and V), in addition to age, sex, DM, hypertension, COVID-19 infection, use of nephrotoxic drugs, and recent surgery. Data were collected through electronic medical record review. Urine output was not included as a criterion for CA-AKI in this study, primarily because the majority of patients did not collect urinary output data. As this was an observational study, it was not possible to ask for urinary output collection for all patients, and the use of serum creatinine changes allowed for a more standardized and practical approach in this context.

We defined nephrotoxic medications as NSAIDs (acetylsalicylic acid, ibuprofen, naproxen, diclofenac), diuretics (furosemide), angiotensin-converting enzyme inhibitors (enalapril, captopril, lisinopril), angiotensin receptor blockers (losartan, valsartan, candesartan), antibiotics (gentamicin, tobramycin, amikacin, streptomycin, neomycin, rifampicin, sulfadiazine, vancomycin, amphotericin B), and antivirals (acyclovir, indinavir, foscarnet)^11,12,13,14^. Surgeries performed within 30 days prior to contrast-enhanced CT were considered a risk factor. Only surgeries that involved large blood volume mobilization, such as cardiac surgery and abdominal laparatomy, were considered as risk factors.

The ICM used during the study was iopamidol, a non-ionic monomer, low-osmolar contrast medium, with iodine concentration of 300 mg I/mL. The injection technique of the contrast material followed the standard hospital protocol, with an average volume of 80 mL per patient (mean volume/weight ratio of 1.2 ml/kg). Deviations from the protocol (i.e., higher volumes) occurred in less than 2% of the patients. Prophylactic measures were not routinely used, as they are not standardized in our institution due to their apparent lack of efficacy.

The population sample to find a between-group difference of 6%, with an alpha of 0.5 and a power of 80%, was calculated to be a minimum of 401 patients per group. Statistical analyses were performed using the R computing program (version 4.0.3). Dichotomous variables are displayed as counts with percentages. Categorical variables were analyzed using Chi-squared test and are displayed as counts and relative frequencies (%). Continuous data were compared using Kruskal-Wallis test and presented as median (25th percentile – 75th percentile). For the linear regression model, continuous variables were adjusted using restricted cubic splines (5 knots per continuous variable) based on variable quantiles established by Harrell and Levy^15^. Due to non-normality, creatinine values were log-adjusted. P-values <0.05 were considered significant.

This study was approved by the Institutional Review Board of Hospital de Clínicas de Porto Alegre (protocol number CAAE 34985220700005327) and conducted in accordance with the Declaration of Helsinki and the Good Clinical Practice guidelines. The researchers ensured the privacy, confidentiality, and anonymity of the data.

There was no funding source for this study.

## Results

A total of 1235 patients were screened from December 2020 to March 2021. After exclusion of ineligible patients (i.e., with missing data), 1003 patients were included in the analyses (489 in the control group and 514 in the exposed group). Individual characteristics are presented in Table 1. There was a statistically significant difference in baseline creatinine (median 0.99 mg/dL in control vs 0.84 mg/dL in exposed, p < 0.001) and age (median 63 in control vs 60 years in exposed, p = 0.009) between groups. Patients in the control group had a significantly higher prevalence of CKD, hypertension, coronary artery disease and congestive heart failure, cerebrovascular disease, peripheral arterial disease, and SARS-CoV-2 infection. Conversely, patients in the contrast-exposed group had a significantly higher prevalence of cancer and recent surgery.

**Table 1:** Baseline population characteristics

Characteristic	Control group (N = 489)	Contrast-exposed (N = 514)	Overall (N = 1003)	p-value
Sex				0.8
Male	247 (51%)	254 (49.4%)	501 (50.0%)	
Female	242 (49%)	260 (50.6%)	502 (50.0%)	
Age	63 (50-73)	60 (46-70)	62 (49-72)	0.09
Pre-CT Scr	0.99 (0.74-1.64)	0.84 (0.70-1.21)	0.91 (0.71-1.32)	<0.001
Pre-CT eGFR	71 (40-94)	86 (57-100)	79 (50-98)	<0.001
Post-CT Scr	0.99 (0.73-1.57)	0.79 (0.67-1.13)	0.87 (0.69-1.29)	<0.001
Post-CT eGFR	72 (39-96)	88 (62-103)	82 (50-100)	<0.001
CKD				<0.001
No CKD	383 (78.32%)	467 (90.9%)	850 (84.74%)	
IIIa	38 (7.77%)	17 (3.3%)	55 (5.48%)	
IIIb	32 (6.54%)	13 (2.5%)	45 (4.48%)	
IV	28 (5.72%)	16 (3.1%)	44 (4.38%)	
V	8 (1.63%)	1 (0.2%)	9 (0.9%)	
Diabetes mellitus 2				0.2
No	434 (88.8%)	469 (91.2%)	903 (90.0%)	
Yes	55 (11.2%)	45 (8.8%)	100 (10.0%)	
Hypertension				<0.001
No	219 (44.8%)	305 (59.3%)	524 (52.2%)	
Yes	270 (55.2%)	209 (40.7%)	479 (47.8%)	
CAD & CHF				<0.001
No	361 (73.8%)	438 (85.2%)	799 (79.7%)	
Yes	128 (26.2%)	76 (14.8%)	204 (20.3%)	
CVD				<0.001
No	337 (68.9%)	463 (90.1%)	800 (79.8%)	
Yes	152 (31.1%)	51 (9.9%)	203 (20.2%)	
Cancer				<0.001
No	416 (85.1%)	356 (69.3%)	772 (76.9%)	
Yes	73 (14.9%)	158 (30.7%)	231 (23.1%)	
ACE inhibitors				0.2
No	406 (83.0%)	443 (86.2%)	849 (84.6%)	
Yes	83 (17.0%)	71 (13.8%)	154 (15.4%)	
ARB				0.3
No	465 (95.1%)	479 (93.2%)	944 (94.1%)	
Yes	24 (4.9%)	35 (6.8%)	59 (5.9%)	
Furosemide				0.06
No	397 (81%)	441 (85.7%)	838 (83.5%)	
Yes	92 (19%)	73 (14.3%)	165 (16.5%)	
Antibiotics				0.5
No	432 (88.3%)	445 (86.6%)	877 (87.4%)	
Yes	57 (11.7%)	69 (13.4%)	126 (12.6%)	
Surgery				0.002
No	441 (90.2%)	429 (83.5%)	870 (86.7%)	
Yes	48 (9.8%)	85 (16.5%)	133 (13.3%)	
Cirrhosis				>0.9
No	473 (96.7%)	496 (96.5%)	969 (96.6%)	
Yes	16 (3.3%)	18 (3.5%)	34 (3.4%)	
Immunosuppressants				0.024
No	462 (94.5%)	501 (97.5%)	963 (96.0%)	
Yes	27 (5.5%)	13 (2.5%)	40 (4.0%)	
NSAIDs				>0.9
No	483 (98.8%)	509 (99.0%)	992 (98.9%)	
Yes	6 (1.2%)	5 (1.0%)	11 (1.1%)	
Antivirals				>0.8
No	454 (92.8%)	480 (93.4%)	34 (6.6%)	
Yes	35 (7.2%)	934 (93.1%)	69 (6.9%)	
Single kidney				0.7
No	486 (99.4%)	512 (99.6%)	998 (99.5%)	
Yes	3 (0.6%)	2 (0.4%)	5 (0.5%)	
Transplant kidney				0.4
No	470 (96.1%)	500 (97.3%)	970 (96.7%)	
Yes	19 (3.9%)	14 (2.7%)	33 (3.3%)	
Coronavirus infection				<0.001
No	428 (87.5%)	488 (94.9%)	916 (91.3%)	
Yes	61 (12.5%)	26 (5.1%)	87 (8.7%)	
PAD				<0.001
No	401 (82.0%)	473 (92.0%)	874 (87.1%)	
Yes	88 (18.0%)	41 (8.0%)	129 (12.9%)	

The SCr variation from baseline to follow-up within 18 to 48h post-CT scan was not significantly different between groups, either using the absolute creatinine change criterion or the relative one (Table 2). The percentage of patients that reached the endpoint of CA-AKI according to each criterion was different, but consistently lower in the group that underwent the enhanced CT scan: absolute SCr increase of 12.4% vs 10.1% (without and with contrast, respectively) and relative SCr increase of 5.3% vs 3.8% (without and with contrast, respectively). The incidence of CA-AKI was also stratified by CKD group, showing similar results (Tables 3 and 4). The overall incidence of CA-AKI according to each criterion was as follows: 11.3% in the absolute SCr increase criterion vs 4.5% in the relative SCr increase criterion.

**Table 2:** Absolute and relative creatinine change per group

Groups	Without contrast (N = 489)	With contrast (N = 14)	p-value
Absolute creatinine change (mg/dL)	–0.01 (–0.15 – 0.11)	–0.02 (–0.12 – 0.06)	0.3
Relative creatinine change (%)	–1 (–14 – 11)	–3 (–13 – 7)	0.2

Median and interquartile range.

**Table 3:** Number and percentage of patients that reached the main endpoint by the absolute creatinine increase criterion

CKD Group	Without contrast (N = 489)	With contrast (N = 514)
Overall	61/489 (12.4%)	52/514 (10.1%)
eGFR > 60	15/289 (5.2%)	25/363 (6.8%)
CKD IIIa	7/54 (12.9%)	12/84 (14.2%)
CKD IIIb	11/56 (19.6%)	5/30 (16.6%)
CKD IV	21/60 (35.0%)	8/29 (27.5%)
CKD V	7/30 (23.3%)	2/8 (25.0%)

Categorical variables reported as count and percentages. eGFR: estimated glomerular filtration rate; CKD: chronic kidney disease.

**Table 4:** Number and percentage of patients that reached the main endpoint by the relative creatinine increase criterion

CKD group	Without contrast (N = 489)	With contrast (N = 514)
Overall	26/489 (5.3%)	20/514 (3.8%)
eGFR > 60	10/289 (3.4%)	15/363 (4.1%)
CKD IIIa	4/54 (7.4%)	3/84 (3.5%)
CKD IIIb	3/56 (5.3%)	2/30 (6.6%)
CKD IV	9/60 (15.0%)	1/29 (3.4%)
CKD V	0/30 (0.0%)	0/8 (0.0%)

Categorical variables reported as count and percentages. eGFR: estimated glomerular filtration rate; CKD: chronic kidney disease.

**Figure 1.**
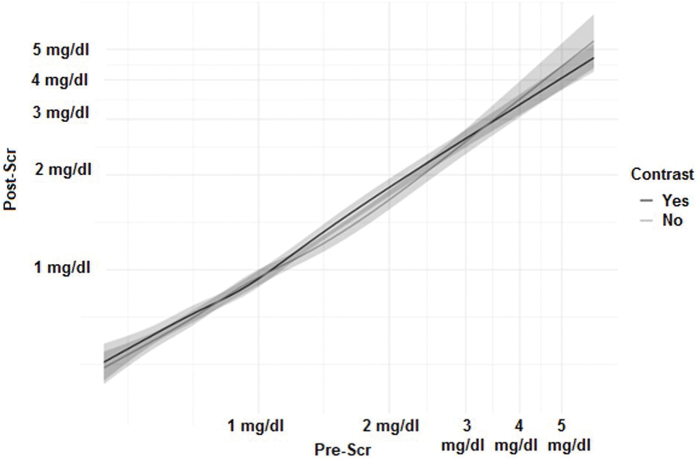
Regression model of the correlation between pre-Scr and post-Scr, adjusted for sex, age, diabetes, hypertension, furosemide, and NSAIDs use. Scr: serum creatinine. The adjacent gray zone represents the 95% confidence interval. The axes are log-adjusted to improve visualization.

**Figure 2.**
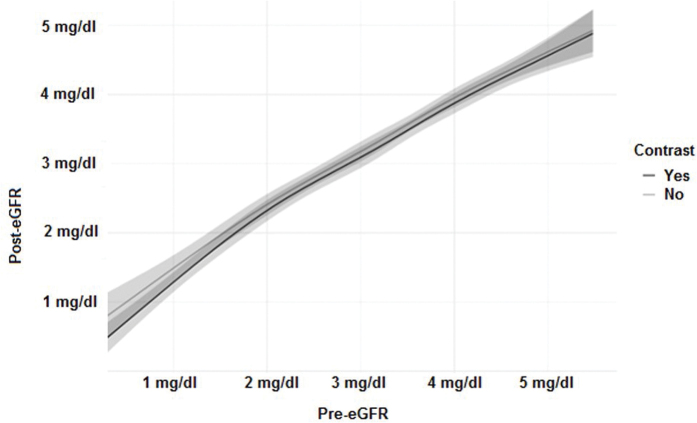
Regression model of the correlation between pre-eGFR and post-eGFR, adjusted for sex, age, diabetes, hypertension, furosemide, and NSAIDs use. eGFR: estimated glomerular filtration rate. The adjacent gray zone represents the 95% confidence interval.

A linear regression model was used to estimate the association between contrast use and post-CT renal function. After adjusting for multiple baseline factors, we found no influence of contrast use on post-CT renal function, whether assessed by SCr levels (p = 0.72) or by estimated glomerular fraction (eGFR) variation (p = 0.13). There was no significant interaction between baseline kidney function and contrast use (p = 0.98 and p = 0.37, respectively), indicating that baseline kidney function was able to predict post-CT renal function regardless of contrast use (Figures 1 and 2).

## Discussion

The present prospective cohort study revealed several crucial findings. First, it demonstrated that contrast use in CT scans was not associated with worsening renal function. Instead, renal function decline is a common occurrence in tertiary hospitals, which likely reflects the severity of cases among these hospitalized patients rather than the accumulation of damage due to ICM use. Additionally, our study indicates the need for more data regarding CA-AKI to determine if it still occurs with newer ICMs and, if so, its true incidence. Finally, we observed a significant disparity in AKI prevalence depending on the KDIGO criterion used, raising the question of which criterion should be employed and whether newer metrics that more accurately reflect AKI should be developed.

The results of this prospective cohort are in line with recent publications by McDonald et al.^5–7^, showing that the group exposed to contrast media did not experience higher rates of AKI, even in patients with CKD. After linear regression and adjustment for multiple confounders, there was no interaction between contrast use and post-CT kidney function. In fact, the only factor truly related to post-CT kidney function was baseline kidney function. Garfinkle et al.^16^ used a different method for assessing CA-AKI incidence, in which they used the creatinine trend of the preceding 24 hours as baseline and defined new AKI as a creatinine level increase at a faster pace than that of baseline. With this methodology, they demonstrated a minimal risk of contrast medium-induced nephropathy and an insignificant risk of requiring long-term dialysis due to contrast media use.

Moreover, we found that AKI is common in tertiary hospitals, occurring in approximately 10% of the patients of our cohort. Our findings emphasize the need for constant monitoring and preemptive identification of patients at risk of AKI, since it has been shown to be frequent and an independent factor for in-hospital mortality, with more severe declines correlating with worse outcomes^17,18^.

Defining AKI remains a matter of intense debate. In our cohort, simply switching from one KDIGO criterion to the other resulted in the rate of AKI varying from 11.3% to 4.5% (absolute vs relative increase criteria, respectively). These differences raise questions about whether creatinine should still be used as a surrogate measure of renal function, since it takes a few days to achieve a steady state^19^, and whether we should continue to use such small absolute variations as a definition of AKI^20^. Lin et al.^20^ assessed the false-positive rate when using KDIGO definitions in hospitalized patients by assessing creatinine level at least four times within a 48-hour interval. The overall false-positive rate in his research was 8%, which increased to 30.5% in patients with a baseline SCr ≥ 1.5 mg/dL. The ideal metric for defining AKI, therefore, remains a topic of discussion.

Our study has several limitations. First, it was an observational study, and as such, had baseline differences between the two groups. The analyses were adjusted for the risk factors most commonly cited in the literature, but there are always unknown factors that are not considered in adjustments. Second, we used only two creatinine set points for most patients, which raises questions about whether the first value truly represented the baseline level and if the second value was indeed indicative of AKI or simply a variation of the method or a false-positive. Third, we were unable to retrieve data about CT scan indication and whether contrast recipients received more preventive measures, such as intravenous hydration, although such prophylactic measures are not standardized and thus rarely used in this hospital. Nevertheless, the incidence of AKI was numerically more common in patients not exposed to contrast, decreasing the impact of this limitation. Finally, due to the observational nature of the study, we were unable to collect urinary output data for most patients and thus had to focus on serum creatinine as the primary criterion for defining AKI.

In the context of decision-making and propedeutic conduct, it is crucial to rationally consider the use of imaging exams with iodinated contrast, particularly when there is a primary indication with an obvious diagnostic benefit. The trade-off between the diagnostic benefits of contrast-enhanced imaging and the potential risk of developing AKI related to iodinated contrast should always be considered. It is important to note that current research has not found an association between contrast use and loss of kidney function, which should be taken into account when providing the most accurate and efficient patient care.

Complementing these individualized assessments and adopting a standardized approach to risk stratification and propedeutic conduct can help optimize the use of iodinated contrast in clinical practice. This may include the development and implementation of evidence-based guidelines and protocols that outline the appropriate indications for contrast-enhanced imaging, taking into account patient-specific factors such as age, comorbidities, and baseline kidney function. Furthermore, the adoption of preventive measures, such as adequate hydration and use of lower volumes or less nephrotoxic contrast agents, can help minimize the risk of AKI in high-risk patients. By integrating these strategies into routine clinical practice, physicians can ensure that the decision to use iodinated contrast is based on a comprehensive and rational evaluation of the potential benefits and risks. In light of the findings from this research, which showed no association between contrast use and loss of kidney function, it is suggested that physicians can safely use contrast media to improve patient care and outcomes, without being concern with contrast-induced AKI.

In conclusion, our findings indicate that the variation in post-CT creatinine values is unrelated to the use of contrast media, challenging the current paradigm of CA-AKI, and suggesting that the excessive fear of contrast media use is unwarranted in patients without CKD classes IV and V. Furthermore, it highlights that the AKI definition remains controversial, and that renal function decline is common in hospitalized patients, occurs independently of contrast use, and ultimately correlates with poorer outcomes.
